# Therapeutic Potential of Mesenchymal Stem Cell-Derived Extracellular Vesicles to Treat PCOS

**DOI:** 10.3390/ijms241311151

**Published:** 2023-07-06

**Authors:** Hang-Soo Park, Esra Cetin, Hiba Siblini, Jin Seok, Hiba Alkelani, Samar Alkhrait, Farzana Liakath Ali, Mohammad Mousaei Ghasroldasht, Analea Beckman, Ayman Al-Hendy

**Affiliations:** Department of Obstetrics and Gynecology, University of Chicago, Chicago, IL 60637, USA; hspark06@bsd.uchicago.edu (H.-S.P.); ecetin1@hurleymc.com (E.C.); hsiblini@bsd.uchicago.edu (H.S.); jjin8977@gmail.com (J.S.); halkelani@bsd.uchicago.edu (H.A.); skhrait@bsd.uchicago.edu (S.A.); fliakathali@bsd.uchicago.edu (F.L.A.); mmghasroldasht@bsd.uchicago.edu (M.M.G.); analeabeckman@uchicago.edu (A.B.)

**Keywords:** mesenchymal stem cell, extracellular vesicles, exosome, polycystic ovary syndrome, intravenous injection, intraovarian injection, fertility

## Abstract

Polycystic ovary syndrome (PCOS) is known as the most common endocrine disorder in women. Previously, we suggested that human mesenchymal stem cells (MSCs) can reverse the PCOS condition by secreting factors. Here, we evaluated the therapeutic capability of MSC-derived extracellular vesicles (EVs), also known as exosomes, in both in vitro and in vivo PCOS models. Exosomes were used to treat androgen-producing H293R cells and injected in a mouse model through intraovarian and intravenous injection into a letrozole (LTZ)-induced PCOS mouse model. We assessed the effects of the exosomes on androgen-producing cells or the PCOS mouse model by analyzing steroidogenic gene expression (quantitative real-time polymerase chain reaction (qRT–PCR)), body weight change, serum hormone levels, and fertility by pup delivery. Our data show the therapeutic effect of MSC-derived EVs for reversing PCOS conditions, including fertility issues. Interestingly, intravenous injection was more effective for serum glucose regulation, and an intraovarian injection was more effective for ovary restoration. Our study suggests that MSC-derived exosomes can be promising biopharmaceutics for treating PCOS conditions as a novel therapeutic option. Despite the fact that we need more validation in human patients, we may evaluate this novel treatment option for PCOS with the following clinical trials.

## 1. Introduction

Polycystic ovary syndrome (PCOS) is known as the most common endocrine disorder which is reported in around 15–18% of reproductive-age women [1,2]. The disorder is characterized by hyperandrogenism, ovulatory dysfunction, and polycystic ovarian morphology [1]. These factors contribute to anovulation and infertility [3,4]. Many women with PCOS also reported metabolic aberrations, such as insulin resistance, dyslipidemia, and increased cardiovascular risk factors including hypertension. Especially, it is reported that it is often worsened by concomitant adiposity in the visceral compartment. These factors can prompt women to abnormal glucose tolerance, cardiovascular disease and, frequently, metabolic syndrome [5,6,7]. Currently, there is no effective medication for a fundamental PCOS cure, and available treatment options aim to manage the symptoms of the condition. These may include medications such as hormonal contraceptives, insulin sensitizers, and fertility drugs. Lifestyle changes, such as exercise and dietary modifications, can also be beneficial in managing the symptoms of PCOS [8,9].

While the exact causes of PCOS are not fully understood, it is thought to be a multifactorial disorder that involves genetic, environmental, and lifestyle factors. In PCOS, molecular markers of inflammation can be triggered by increased concentrations of glucose and saturated fat in circulation. In addition, it is highly correlated with insulin resistance and hyperandrogenemia [10,11,12,13]. Proinflammatory stimuli can stimulate theca-cell androgen production and the expression of enzymes responsible for producing androgen in vitro [14]. Therefore, increased intraovarian chronic inflammation is an important factor in the pathogenesis of PCOS [14]. In the last decade, many studies within the literature have established the robustness of the immunosuppressive and anti-inflammatory capabilities of human mesenchymal stem cells (MSCs) [15,16,17,18].

MSCs are a widely used adult stem cell type that can be isolated from various tissues in the body, including bone marrow, adipose tissue, and umbilical cord. They are multipotent stem cells and have immunomodulatory and anti-inflammatory properties [19]. Several published papers have reported the therapeutic effect of MSCs or their secreting factors (i.e., conditioned media) in various diseases, including neurological disorders [20], cardiac ischemia [21], diabetes [22], bone and cartilage diseases [23], liver injury or fibrosis [24], spinal cord injury, and wound healing [25]. Over the last decade, extensive research has been conducted on the mechanisms driving the immunosuppressive and anti-inflammatory effects of MSCs [26,27]. Secreted factors, such as IL-10, TGF-B1, and extracellular vesicles containing various miRNAs, are potential primary mediators of such therapeutic effects [15,28]. 

Recent studies reported that the regenerative potential of MSCs is mainly achieved through the paracrine activity, including exosome. Exosomes are small extracellular vesicles (EVs) which are secreted by cells and contain various biomolecules, such as miRNA and peptide. Exosome can deliver those therapeutic biomolecules to damaged or malfunctional cells to restore the tissue function [29]. MSCs can migrate toward damaged tissues inherently [30,31,32] and secrete various bioactive molecules, such as exosomes, with a cargo that has angiogenic, antiapoptotic, antifibrotic, anti-inflammatory, and immunosuppressive effects [15,33,34,35]. In addition, the safety of exosome-based therapy has already been reported in several papers. A published paper reported that MSC-derived exosomes are expected to replace stem cells as a new therapeutic option for a regenerative as a cell-free approach [36]. Many current and completed clinical trials registered in the www.ClinicalTrials.gov (accessed on 12 June 2023) database also demonstrate the regenerative potential and safety of exosome-based therapy in various diseases [37,38]. Through those studies, exosomes are considered to be one of the promising key molecules which mediate the therapeutic effect of MSCs without safety concerns. Many current and completed clinical trials have already reported the therapeutic benefits of MSC-derived exosomes in various conditions, such as graft vs. host disease (GVHD), end-stage renal disease (grade III-IV CKD), and atrophic acne scars [37,38,39]. Most recently, during the COVID-19 pandemic, multiple trials were performed to test the therapeutic potential of exosome treatment in severe COVID-19 cases [40,41,42]. Although there are many studies and clinical trials using MSC-derived exosomes in various diseases, it is not easy to find a clinical trial using exosomes for reproductive disorders such as PCOS. To increase the chance of success in a clinical trial using MSC-derived exosome for PCOS treatment, we need to confirm and compare their therapeutic potential in a PCOS animal model.

Several studies have also demonstrated the positive therapeutic effect of MSCs in PCOS animal models via immunomodulation [43,44]. In a recent study, our group reported that the MSC secretome regulates androgen production in vitro in H295R cells, a human-ovarian-theca-like cell line [45,46,47], and reverses PCOS-related morbidities (insulin resistance and infertility) in a letrozole-induced PCOS mouse model [48]. Secreted factors derived from MSCs can regulate inflammation, fat metabolism, and ovarian function in a PCOS animal model [48,49]. However, these previous studies used whole secretome and did not address the effect of the purified component from MSCs in a PCOS animal model. In addition, as well as other biopharmaceutical materials, the suitability of an injection route for exosome also needs to be confirmed before applying exosome-based therapy for PCOS treatment.

In this study, we report the therapeutic effect of purified MSC-derived exosome treatment via intravenous and intraovarian injection in a PCOS mouse model. We tested the therapeutic potential of MSC-derived exosomes in an in vitro cell model by comparing androgen-producing gene expression. We also injected MSC-derived exosomes directly into the ovary or intravenously in a PCOS mouse model. We compared several PCOS-related parameters, such as fertility and metabolism, in LTZ-induced PCOS mice.

## 2. Results

### 2.1. Effect of MSC-Derived Exosomes on Androgen-Producing Cells

**Figure 1:**
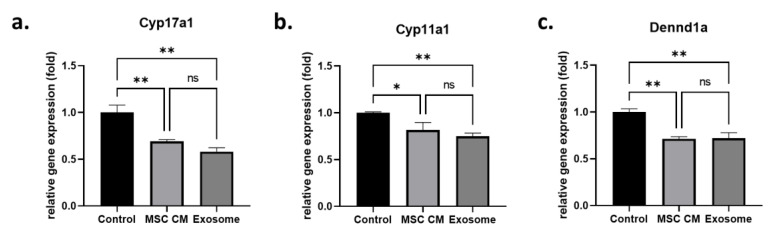
Effect of MSC-derived exosomes on androgen production in an in vitro cell model. Relative gene expression levels of Cyp17a1 (**a**), Cyp11a1 (**b**), and Dennd1a (**c**) in H295R cells in the control group (control), MSC-CM-treated group, (MSC CM) and MSC-derived exosome-treated group (exosome). Data are presented as the mean ± SD (n = 3, significance level * *p* < 0.05, ** *p* < 0.005, ns: not significant).

After treating H295R cells with MSC-derived conditioned media (MSC CM) or purified MSC-derived exosomes, we analyzed the gene expression levels of androgen-producing genes such as Cyp17a1, Cyp11a1, and DENND1a. We found that Cyp17a1 gene expression was significantly decreased in both MSC CM (0.69 ± 0.02-fold)- and exosome (0.58 ± 0.04-fold)-treated H295R cells (Figure 1a). In addition, there was no significant difference between MSC-CM-treated cells and exosome-treated cells (*p* = 0.16). Another cytochrome P450 family gene, Cyp11a1, also showed similar results (Figure 1b). Both MSC-CM-treated cells and exosome-treated cells showed significantly decreased Cyp11a1 gene expression (0.82 ± 0.08-fold and 0.75 ± 0.03-fold, respectively) compared to that in the untreated control group, and there was no significant difference between expression with MSC CM treatment and exosome treatment (*p* = 0.37). The effect of the exosomes on androgen-producing genes was confirmed with another androgen-producing gene, DENND1a (Figure 1c). Similar to the other genes, DENND1a expression was significantly downregulated in MSC-CM-treated cells (0.71 ± 0.02-fold) and exosome-treated cells (0.72 ± 0.06-fold). 

### 2.2. MSC-Derived Exosome Treatment Reverses Metabolism in a PCOS Mouse Model

**Figure 2:**
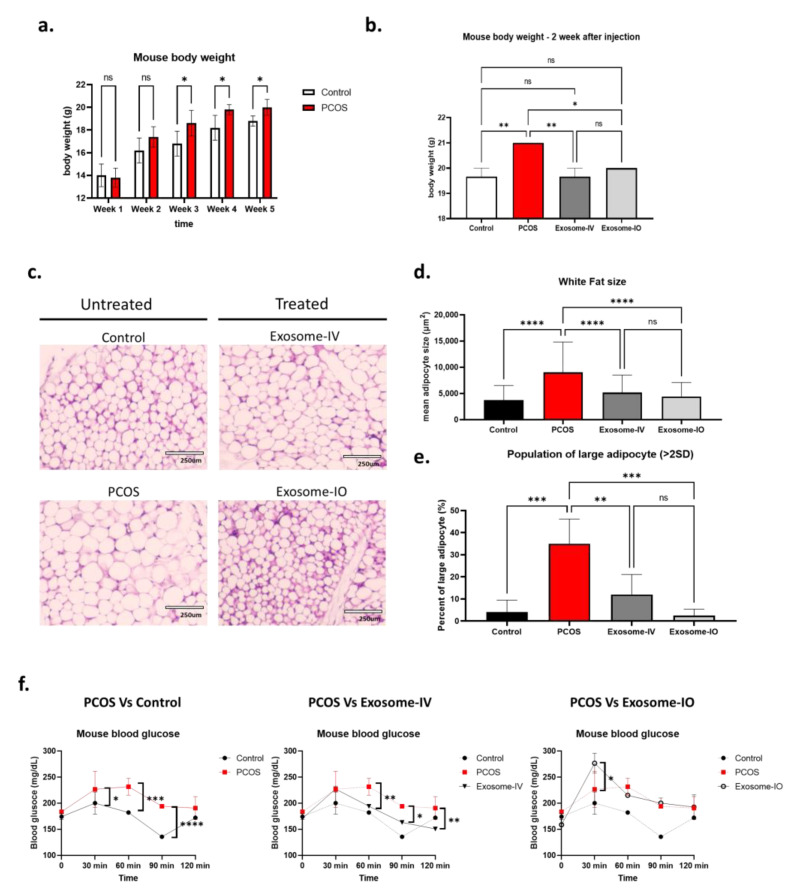
Regulation of metabolic parameters by MSC-derived exosome treatment in a PCOS mouse model. (**a**) Mouse body weight changes during PCOS induction (n = 5/groups). (**b**) Comparison of mouse body weights between groups two weeks after exosome treatment (n = 3/group). (**c**) Representative image of mouse gonadal white adipose tissue morphology (H&E staining). (**d**) Average size of adipocytes in white fat tissue. (**e**) Percentage of large adipocytes from the whole adipocyte population. (**f**) Glucose tolerance test after intraperitoneal (i.p.) glucose injection (n = 3/group). Data are presented as the mean ± SD (significance level * *p* < 0.05, ** *p* < 0.005, *** *p* < 0.0005, **** *p* < 0.0001; ns: not significant).

We confirmed PCOS induction in a mouse model by changes in body weight until week 5 (Figure 2a). After induction (at week 5), PCOS mice showed significantly increased body weight (20.0 ± 0.7 g) compared to that of the control group mice (18.8 ± 0.45 g). We randomly divided PCOS mice into three groups: untreated PCOS group (PCOS), exosome treatment group through intravenous injection (exosome-IV), and exosome treatment group through intraovarian injection (exosome-IO). Two weeks after treatment, we found that the body weight of treated mice was reversed compared to the healthy control level (Figure 2b). Both the exosome-IV group (19.7 ± 0.6 g) and exosome-IO group (20.0 ± 0.0 g) showed significantly lower body weights than those of the PCOS group (21.0 ± 0.0 g), and this same trends were apparent with the control group (19.7 ± 0.6 g). We next observed the size of adipocytes in white adipose tissue (Figure 2c–e). By histological H&E staining, we found that the average size of adipocytes was significantly decreased in the exosome-IV group (5198 ± 3306 µm^2^) and exosome-IO group (4424 ± 2660 µm^2^), while the PCOS group still showed enlarged adipocytes (9030 ± 5797 µm^2^). We also compared the population of large adipocytes, which was defined as larger than 95% (>mean + 2SD) of a healthy mouse adipocyte (3711 ± 2788 µm^2^). Our data revealed that the untreated PCOS group had a significantly higher population of large adipocytes (35.0 ± 11.2%), but the population was restored to the healthy range in both the exosome-IV group (12.0 ± 9.1%) and exosome-IO group (2.5 ± 2.8%), without a significant difference from the healthy control (Figure 2e). At last, we measured glucose levels at 30, 60, 90, and 120 min after glucose injection (Figure 2f). In our data, blood glucose levels were higher in the PCOS group than in the healthy control group, but the exosome-IV group showed decreased blood glucose levels, and the trend was the same as that of the healthy mouse group. The exosome-IO group did not show any enhancement in blood glucose level compared to untreated PCOS. 

### 2.3. MSC-Derived Exosomes Restore Ovarian Function in a PCOS Mouse Model

**Figure 3:**
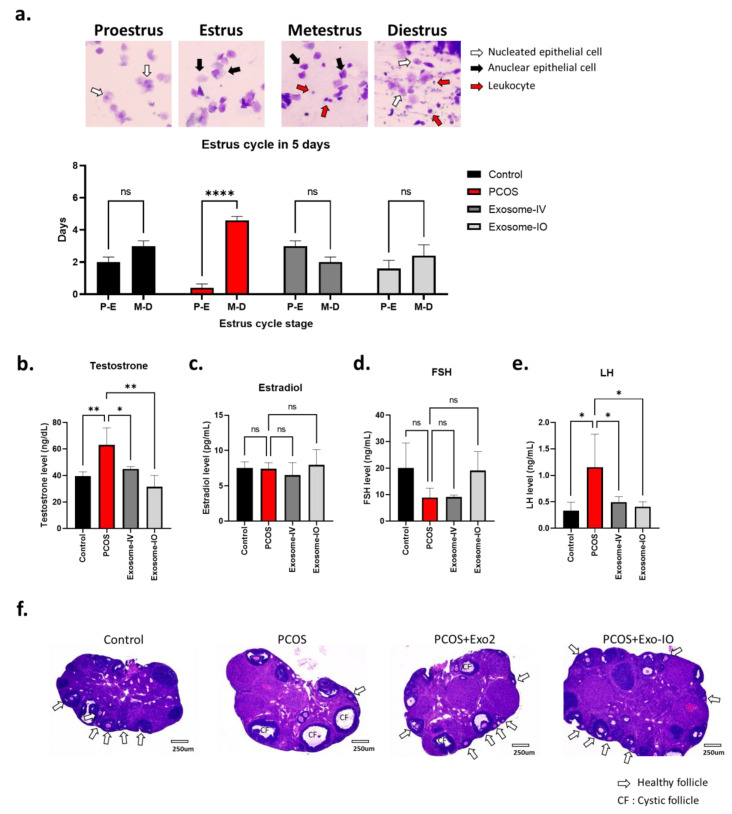
Restoration of ovarian function by MSC-derived exosome treatment in a PCOS mouse model. (**a**) Comparison of the frequency of the ovulating stage (proestrus-estrus, P-E) and nonovulating stage (metestrus-diestrus, M-D) over 5 days (n = 5/group). (**b**–**e**) Serum hormone level comparison between the control, PCOS, exosome-IV, and exosome-IO groups (n = 3/group). (**f**) Representative image of mouse ovaries in the control, PCOS, exosome-IV, and exosome-IO groups (H&E staining). White arrows indicate healthy follicles, and cystic follicles are highlighted as CFs. Data are presented as the mean ± SD (n = 3, significance level * *p* < 0.05, ** *p* < 0.005, **** *p* < 0.0001; ns: not significant).

Our data showed that the estrous cycles of PCOS mice were arrested in nonovulating stages (M-D), while the cycles of healthy mice were evenly distributed in ovulating (P-E) and nonovulating stages (M-D). Interestingly, we found that both the exosome-IV and exosome-IO groups showed more ovulating stages in their cycle than those of the healthy control mice (Figure 3a). Furthermore, we analyzed the serum levels of testosterone, estradiol, FSH, and LH to compare ovarian function (Figure 3b–e). Our data show significantly higher testosterone levels in the PCOS group (63.0 ± 12.9 ng/dL) than in the healthy control group (39.7 ± 3.2 ng/dL), which indicates typical hyperandrogenemia in PCOS conditions. We found that this altered testosterone level was significantly reversed in both the exosome-IV group (45.1 ± 1.7 ng/dL) and exosome-IO group (31.6 ± 8.6 ng/dL) (Figure 3b). The estradiol level was not significantly changed in any group (Figure 3c). The serum FSH level did not change significantly but showed a decreasing trend in the PCOS group. Interestingly, only the exosome-IO group showed an increasing trend compared to the PCOS group (Figure 3d). On the other hand, LH levels showed a significant difference after exosome treatment. The PCOS group showed a significantly higher LH level (1.16 ± 0.62 ng/mL) than that of the healthy control group (0.33 ± 0.16 ng/mL), which is a typical characteristic of PCOS. Our data showed that this altered LH level was significantly reversed in both the exosome-IV group (0.49 ± 0.10 ng/mL) and exosome-IO group (0.41 ± 0.1 ng/mL) (Figure 3e). Our histology data also indicated that ovarian structures were restored by exosome treatment. We performed H&E staining of cross-sections of the mouse ovary to compare tissue morphology (Figure 3f). The PCOS mouse ovary had more cystic follicles (CF) and fewer follicles than those of the healthy control mouse ovary. Mouse ovaries from the exosome-IV and exosome-IO groups showed more follicles and less cystic morphology than those of the untreated PCOS group and looked similar to the healthy mouse ovary. 

### 2.4. MSC-Derived Exosomes Restore Fertility in a PCOS Mouse Model

**Figure 4:**
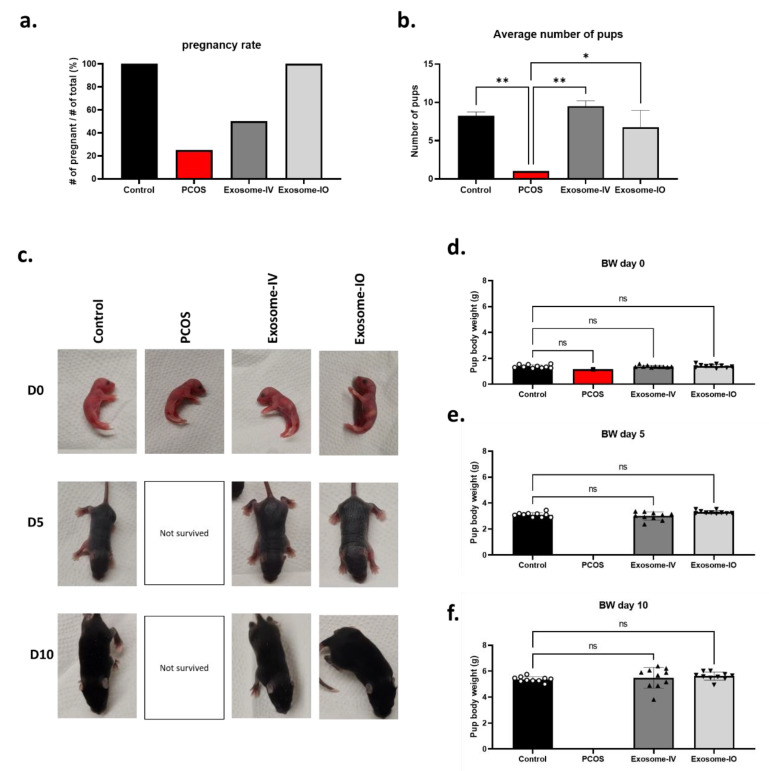
Fertility restoration by MSC-derived exosomes in a PCOS mouse model. (**a**) Pregnancy rate calculation in the control, PCOS, exosome-IV, and exosome-IO groups (n = 4/group). (**b**) Average number of pups in pregnant mice. (**c**) Representative image of newborn pups on day 0, day 5, and day 10. (**d**–**f**) Average body weight of offspring from the control, PCOS, exosome-IV, and exosome-IO groups at day 0, day 5, and day 10. Symbols on the bar graph indicate the actual body weight of each offspring (n = 10/group). Data are presented as the mean ± SD (n = 3, significance level * *p* < 0.05, ** *p* < 0.005; ns: not significant).

In the breeding result, most of the PCOS mice were infertile, and only one out of four (25%) achieved pregnancy, while all the mice became pregnant in the healthy control group (100%). Two out of four of the exosome-IV group mice became pregnant (50%), indicating restored fertility. All mice in the exosome-IO group achieved pregnancy (100%), similar to the healthy control group (Figure 4a). We also compared the average number of pups between groups (Figure 4b). The healthy mouse group delivered 8.3 ± 0.5 pups per litter, while the PCOS mouse group only delivered 1 pup per litter. Both the exosome-IV group (9.5 ± 0.7 pups per litter) and exosome-IO group (6.8 ± 2.2 pups per litter) delivered multiple pups, which demonstrates restored fertility by exosome treatment. Pups delivered from the healthy control, exosome-IV, and exosome-IO groups were active and healthy until 10 days postnatal. On the other hand, pups delivered from the PCOS group could not survive longer than 5 days due to their small litter size (Figure 4c). We measured the body weight of pups (n = 10) at days 0, 5, and 10 to compare the growth rate. We found that there were no differences among pups from the healthy control group, exosome-IV group, and exosome-IO group (Figure 4d–f). 

## 3. Discussion

### 3.1. MSC-Derived Exosomes Mediated Fertility Restoration in a PCOS

In a previous study, we revealed that MSCs can restore fertility in a mouse PCOS model through paracrine effects [48,49,50]. In this study, we report a significant downregulation of the steroidogenesis gene in H295R cells by MSC-derived exosomes. Interestingly, our data show that the effect of purified exosomes was equal to that of whole conditioned media, which indicates that exosomes are the main molecules that regulate androgen production in an in vitro model. Taken together, our in vitro results demonstrated that MSC-derived exosomes are effective as much as the whole secretome to decease androgen synthesis pathways. Based on this result, we conducted an in vivo study using MSC-derived exosomes in PCOS mice, and reported the therapeutic effect of exosome injection through two different routes to restore fertility in a PCOS mouse model.

Through our in vivo study, we first analyzed the effects on ovary function through estrous cycle analysis, serum hormone levels, and ovary morphology. Our data demonstrate that MSC-derived exosome treatment through intravenous or intraovarian injection can successfully restore ovarian function in the PCOS mouse model. After confirming ovarian function, we compared the fertility of exosome-treated mice in a breeding experiment. Two female mice were bred with one healthy male mouse, and the pregnancy rates were calculated by the number of pregnant mice per total female mice. While this LTZ-induced PCOS mouse model has been shown to be infertile [48,49,51], we demonstrated that MSC-derived exosome treatment through both intravenous and intraovarian injection was able to restore fertility, and treated mice delivered healthy pups. Interestingly, intravenous injection was more effective in regulating metabolic aberrations, such as glucose levels, but less effective in restoring fertility compared to intraovarian injection. Our breeding data show that exosome treatment via intravenous or intraovarian injection was successful in restoring fertility in the PCOS mouse model. Previously, there were some PCOS treatment studies using MSCs, but most of those studies did not show breeding data [43,44]. Our group reported restored fertility after MSC secretome treatment in PCOS mice, but that study did not use purified exosome [48]. In addition, according to a recent meta-analysis study, research using MSC-derived exosome for PCOS animal model treatment was not identified [52]. Therefore, our data provide unique information regarding the therapeutic effect of MSC-derived exosomes to achieve pregnancy in the PCOS condition. 

### 3.2. Metabolism Regulation by MSC-Derived Exosome Treatment

Our in vivo experimental data show that both intravenous and intraovarian injections of exosomes were effective in reversing several PCOS-related metabolic abnormalities in our PCOS mouse model. To analyze metabolic outcomes under PCOS conditions, we first measured body weight changes and next observed the size of adipocytes in white adipose tissue. We also analyzed another metabolic outcome, that is, glucose tolerance. Interestingly, the exosome-IO group did not show any enhancement in blood glucose level even though this group showed promising results in body weight change and adipocyte size. Our results indicate that MSC-derived exosome treatment through an intravenous or intraovarian route is promising for regulating PCOS-related obesity. However, it also indicates that intravenous injection is a more promising option to regulate blood glucose levels in PCOS conditions. The mechanism causing the difference between intraovarian injection and intravenous injection is not clear at this moment. It is known that metabolic aberrations, such as altered glucose levels and insulin resistance, are mainly regulated by adipose and liver tissue, not the ovary [53,54,55]. In our previous study, we found that locally injected MSCs did not migrate into other tissue, and the majority of injected cells stayed within injected tissue [56]. In another published paper, researchers showed that fluorescence-labeled nanoparticles were detected in several major tissues in intravenous injection, while they were only detected in tumor tissue in intratumor local injection [57]. Based on those published papers, we assume that the higher efficacy of intravenous injection in metabolic regulation is due to the higher distribution rate of injected exosomes to the liver and adipose tissue. Proving the scientific evidence of this mechanism of action might be an interesting topic for future study.

### 3.3. Mechanism of MSC-Derived Exosome in PCOS Treatment

In our invitro study, we used the androgen-producing H295R cell line, called theca-like cells, to confirm androgen-producing capacity changes. Our results demonstrate that MSC-derived exosomes can decrease androgen-producing pathways in H295R cells. This also indicates that MSC-derived exosomes can regulate and exceed the andro-gen level in PCOS patients. According to a published paper, androgen production is triggered by inflammation [12]. In our previous paper, we reported that anti-inflammatory cytokines, such as IL-10, are considered the main molecule that reverses the PCOS condition by regulating inflammation in the PCOS mouse model [48]. In this previous study, we also observed not only restored fertility but also metabolic changes, such as glucose level and adipose tissue morphology. Similar to our previous report, we consider IL-10 to be a key molecule in regulating the PCOS condition through exosome treatment. Recent studies have reported that in many in vitro and in vivo systems, the majority of IL-10 cytokines are encapsulated in exosomes when secreted [58]. It has been shown that IL-10 cytokines are significantly less stable in their free form than in their exosome-encapsulated form. Exosome encapsulation may protect cytokines and help direct cytokine delivery to target cells. In prior sonication experiments, it was found that the biological activity of exosome-encapsulated IL-10 was the same as that of the free cytokine itself, suggesting that IL-10 contributes to exosome activity when encapsulated. Once the exosome arrives at the IL-10 receptors on a cell surface, it likely releases the cytokines at a higher surface concentration than if the cytokines were just released into the extracellular space, thus streamlining cell–cell communication [58]. Therefore, we suggest that exosomal IL-10 is the key molecule to reverse PCOS conditions in exosome-based treatment. 

### 3.4. Benefit of MSC-Derived Exosome Based Treatment

As we already reported, MSCs have a great regulatory effect on the PCOS condition. However, using whole cells, especially allogenic cells from a healthy donor, still causes concern despite the reported immunosuppressive properties, which means they can avoid immune reactions [26]. Recently, many researchers have noted MSC-derived exosomes due to their therapeutic potential as well as accessibility and wide availability [59,60,61]. Moreover, compared with the whole process of preparing and storing stem cells, exosomes offer an excellent feasible alternative as they are more accessible and less expensive to obtain [62,63]. In addition, the clinical trial using exosome-based therapy for reproductive disorder has already been approved. Most recently, our team successfully obtained FDA approval for the first IND study on exosome therapy for a reproductive disorder. The clinical trial is a first-in-human phase 1 pilot trial to examine the safety and efficacy of MSC-derived exosome therapy in patients with premature ovarian insufficiency. It is an open-label, prospective, interventional trial where single-dose exosome therapy will be administered intravenously to patients diagnosed with premature ovarian insufficiency (POI) based on the American Society of Reproductive Medicine guidelines, and enrollment is planned to start soon (IND 28896). For these reasons, among stem cell-based therapy options, exosome therapy is a more stable, safer, and cell-free approach that can be widely applied and studied in regenerative medicine clinical applications [36,37]. 

In this study, we provided further reliable evidence of the therapeutic potential of MSC-derived exosomes in reversing PCOS through several treatment routes. Our study provides preliminary baseline data for future clinical trials using intravenous or intraovarian exosome injection in patients with PCOS. Based on the scientific findings in this study, we are planning to start a clinical trial for PCOS treatment using MSC-derived exosomes. Through our future clinical study, we may verify the therapeutic effect of MSC-derived exosomes in human PCOS patients.

## 4. Materials and Methods

### 4.1. Invitro PCOS Cell Model (Human Adrenocortical Carcinoma Cell Line) Culture

Androgen-producing human adrenocortical carcinoma cells (H295R cells) were used as an in vitro cell model to mimic theca cells from PCOS patients. These cells are an immortalized cell line and have been used in published papers for steroidogenesis and androgen synthesis pathways [45,64,65]. We purchased H295R cells from ATCC (Manassas, VA, USA, cat. no. ATCC^®^ CRL-2128™) and cultured with the recommended protocol. Briefly, T75 cell culture flasks were precoated with attachment factor (Gibco, Billings, MT, USA, cat. no. S-006-100), and H295R cells were cultured in coated T75 flasks (approximately 13,000 cells/cm^2^). DMEM/F12 (Gibco, cat. no. 21041025), 2.5% Nu-Serum (Corning, Corning, NY, USA), and 1% ITS+ Premix supplement (Corning) were used for cell culture media.

### 4.2. Preparation of the MSC-Conditioned Media and MSC-Derived Exosomes

Human-umbilical-cord-derived MSCs were purchased from Roosterbio (Frederick, MD, USA) and were cultured per the manufacturer’s instructions. MSC-conditioned media (MSC CM) were collected when cells (passage 4) reached approximately 80% confluency. Cells were then washed three times with phosphate-buffered saline (PBS) to completely remove serum. Then, cells were cultured for 24 h in DMEM/F12 (Gibco) serum-free media. After incubating for 24 h, we collected conditioned media, centrifuged for 5 min with 500 G at 4 °C to remove the cell debris, and stored at −80 °C for use in future experiments. MSC-derived exosomes were isolated from MSC CM via the PEG-precipitation method using a commercialized reagent (ExoQuick-TC, System Biosciences, Palo Alto, CA, USA). Isolated exosomes were stored at −80 °C for use in further experiments. In the animal study, commercially available exosomes (Vitti lab, Liberty, MO, USA) were used due to the large amount of exosome supply required for the entire experiment.

### 4.3. Treatment of H295R Cells with MSC CM

MSC CM were diluted in basal medium (serum-free) at a 1:1 ratio and were treated to H295R cells for 24 h. The exosomes that were produced from a matched amount of MSC CM were also added to basal media (serum-free) and used to treat H295R cells for 24 h as the exosome group. Cells were collected after incubation for further analysis of steroidogenesis-related gene pathways.

### 4.4. PCOS Mouse Model and Exosome Treatment

The animal experiments were approved by the University of Chicago Animal Care Committee (UC IACUC). The animal protocol entitled “Study for treat female reproductive disease using small animal model” was approved by UC IACUC on 11 March 2020 (approval number 72638). All the animal experiments were performed in compliance with the University of Chicago’s policies and guidelines for the use of laboratory animals. Three-week-old female C57BL/6 mice were purchased from Charles River (Wilmington, MA, USA) for the animal experiment. Mice were housed in an animal facility for at least 72 h under specific pathogen-free conditions. At 3.5 weeks of age, a placebo or 5 mg LTZ pellet (Innovative Research of America, Sarasota, FL, USA), which released LTZ constantly (50 μg/day), were subcutaneously implanted into mice (n = 10/group). We monitored body weight on a weekly basis pre- and post-implantation. Body weight changes were used to monitor the development of PCOS characteristics.

Mice underwent the intravenous injection or intraovarian injection of exosomes (Vitti lab, MI) at five weeks after placebo or LTZ pellet implantation. Mice were anesthetized using 1–4% isoflurane inhalation for intravenous injection. Exosomes (1.5 × 10^8^) were resuspended in 100 µL of PBS and injected through the retro-orbital sinus. For intraovarian injection, mice were treated preoperatively with a single dose of meloxicam (5 mg/kg) and were kept under anesthesia with 1–4% inhalation of isoflurane during the entire procedure. A single midline incision, less than 25 mm, was made on the skin to access both ovaries via the caudal abdominal cavity. A total of 1.5 × 10^8^ particles of exosomes in 10 µL of PBS was injected into both ovaries. The incision was closed by suturing, followed by wiping with a clean disinfectant swab. For further analysis, mice were divided into breeding cages (n = 4 per group) and experimental cages (n = 5 per group). After recovery, breeding cage mice started breeding with male mice (two females with one male). Experimental cage mice underwent a GTT assay two weeks after exosome treatment and were anesthetized to collect serum, white fat tissue, and ovaries. One ovary, as well as a portion of the fat tissue, were fixed in 4% paraformaldehyde and embedded in paraffin for histology analysis.

### 4.5. Glucose Tolerance Test (GTT)

Glucose tolerance testing was performed with mice at 2 weeks after exosome treatment. We removed food in the mice cage for 16 h (5 p.m. to 9 a.m.) for fasting. During the fasting period, we allowed free access to drinking water. Then, mice received an intraperitoneal (i.p.) injection of D-glucose (2.0 g/kg body weight). We measured blood glucose levels at 0, 30, 60, 90, and 120 min following glucose injection using a Bayer glucose monitor (Roche Diagnostics Corp, Indianapolis, IN, USA).

### 4.6. Estrus Cycle Analysis

We recorded the estrous cycle through a daily vaginal swab. We collected vaginal cell samples through daily vaginal swabs and smeared them on the slide glass. Slides were stained with 0.5% crystal violet solution for 1 min. After washing the slide with tap water, we examined each slide based on the ratio of cell types [66]. We compared the ratio of time in an ovulating stage (proestrus and estrus stage, P-E) or a nonovulating stage (metestrus and diestrus stage, M-D).

### 4.7. Serum Hormone Measurements

We collected whole blood from all the groups via cardiac puncture under isoflurane anesthesia. The serum was separated using a centrifuge (2000 G for 15 min) and stored at −80 °C. Serum hormone levels were measured at the University of Virginia Ligand Core Facility. We analyzed serum testosterone (T) and estradiol (E2) levels using an ELISA. We also measured serum luteinizing hormone (LH) and follicle-stimulating hormone (FSH) levels using radioimmunoassay (RIA). The sensitivities of each assay were 10 ng/dL (T), 3 pg/mL (E2), 3 ng/mL (FSH), and 0.04 ng/mL (LH).

### 4.8. Breeding Experiments

One week after exosome treatment, 4 mice were randomly selected per group for the breeding experiment. Two female mice were breed with one male breeder mouse (C57BL/6). The male and female mice were housed together for 10 days for mating. The presence of a sperm plug in the vagina was checked for mating monitoring. We confirmed that most of the female mice showed a sperm plug within 3 days. After delivery, we counted the average number of pups from each female mouse and compared them among treatment groups. At the end of the experiment, we recorded all the delivered pups per group, their body weight, and any morphological anomalies.

### 4.9. Histology Assay

We collected ovaries and fat tissues, fixed in 4% paraformaldehyde, and embedded in paraffin to make formalin-fixed, paraffin-embedded tissue (FFPE tissue). FFPE tissue sections were stained with hematoxylin–eosin (H&E) for microscopy. The histology core of the University of Chicago (Chicago, IL, USA) performed sample processing and staining. Histological analyses were performed using Asperio ImageScope (Leica Biosystem, Wetzlar, Germany).

### 4.10. Statistical Analysis

A two-way ANOVA or nonparametric *T* test (Mann–Whitney test) using GraphPad Prism 9 (GraphPad Software, San Diego, CA, USA) was performed for statical comparisons between groups. All data are presented as the mean ± standard deviation (SD). Statistically significant differences between groups are marked with * (*p* < 0.05), ** (*p* < 0.005), or *** (*p* < 0.0005).

## 5. Conclusions

MSC-derived exosomes are as promising for PCOS treatment as the whole MSC cells from which they are derived. Our data indicate that MSC-derived exosomes are the main molecules that regulate androgen production in an in vitro model and restore fertility in a PCOS mouse model. Both intravenous injection and intraovarian injection showed novel therapeutic potential in PCOS conditions. Intravenous injection shows a more effective outcome in systemic regulation, such as blood glucose control. On the other hand, intraovarian injection shows higher efficacy in restoring ovarian function. Although further clinical trials are needed in our future studies, MSC-derived exosomes can be a promising treatment option for PCOS patients.

## Data Availability

All data are present in the paper and/or the supplementary materials.

## Supplementary Materials

The following supporting information can be downloaded at: https://www.mdpi.com/article/10.3390/ijms241311151/s1.
