# Supplementary material for: Therapeutic Potential of Mesenchymal Stem Cell-Derived Extracellular Vesicles to Treat PCOS
Source: Int J Mol Sci. 2023 Jul 6;24(13):11151. doi: 10.3390/ijms241311151 (PMC10342552; doi:10.3390/ijms241311151)
Supplement: Supplementary file 1 [file ijms-24-11151-s001.zip › ijms-2475287-supplementary.pdf]

Supplementary data

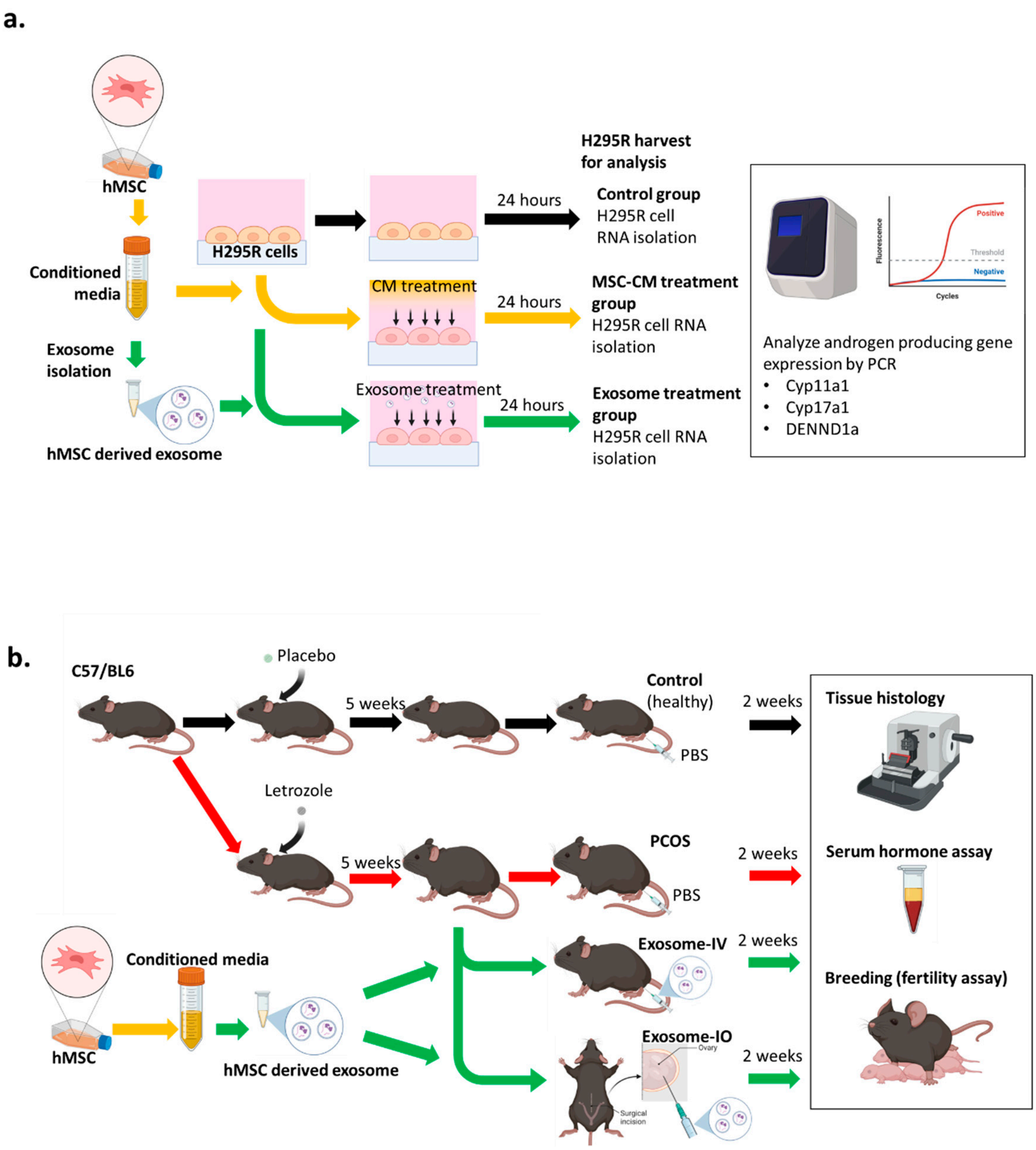

Supplementary Figure S1. Schematic image of the *in vitro* experimental plan (a) and *in vivo* experimental plan (b).
